# Comparison of antioxidant activities among four kinds of Japanese traditional fermented tea

**DOI:** 10.1002/fsn3.442

**Published:** 2016-11-22

**Authors:** Masanori Horie, Kazuhiro Nara, Sakiko Sugino, Aya Umeno, Yasukazu Yoshida

**Affiliations:** ^1^ Health Research Institute (HRI)National Institute of Advanced Industrial Science and Technology (AIST)TakamatsuKagawaJapan; ^2^ Faculty of Human Life SciencesJissen Women's UniversityHinoTokyoJapan

**Keywords:** aerobic fermentation, anaerobic fermentation, antioxidant activity, catechins, fermented tea

## Abstract

Antioxidant activities of four kinds of Japanese traditional fermented tea, Gishi‐cha, Ishizuchi‐kurocha, Awa‐bancha, and Batabatacha, were compared. Antioxidant activity was evaluated by three parameters: copper ion reduction ability, radical trapping ability, and oxygen consumption rate. Processes of fermentation of these fermented teas are different. Goichi‐cha and Ishizuchi‐kurocha are produced by a two‐stage fermentation process, aerobic fermentation and subsequent anaerobic fermentation. Awa‐bancha is produced by anaerobic fermentation. And batabata‐cha is produced by aerobic fermentation. Additionally, unfermented green tea was also employed as control. These tea leaves were extracted by boiling water and measured antioxidant activities. And concentrations of caffeine and catechins were measured in green tea and in the four kinds of fermented tea: Ishizuchi‐kurocha, Goishi‐cha, Awa‐Bancha, and Batabata‐cha. Concentrations of caffeine and catechins were lower in the fermented teas than in green tea. Among the fermented teas, epigallocatechin content was the highest in Ishizuchi‐kurocha, whereas Batabata‐cha hardly contained any epigallocatechin. Goichi‐cha, Ishizuchi‐kurocha, and Awa‐bancha showed antioxidative activity regardless of measurement method. Batabatacha had hardly any antioxidative activity. Among the fermented teas, Ishizuchi‐kurocha had the strongest antioxidant activity. The antioxidative activities of green tea and the four kinds of fermented tea were significantly different among each other (*p *<* *.01). Implication of this study is as follows: although contents of catechins were lower than that of green tea, three kinds of fermented tea showed antioxidative activity comparable to green tea. The results suggest that anaerobic fermentation process is beneficial at least for antioxidative activity.

## 1 Introduction

**Figure 1:**
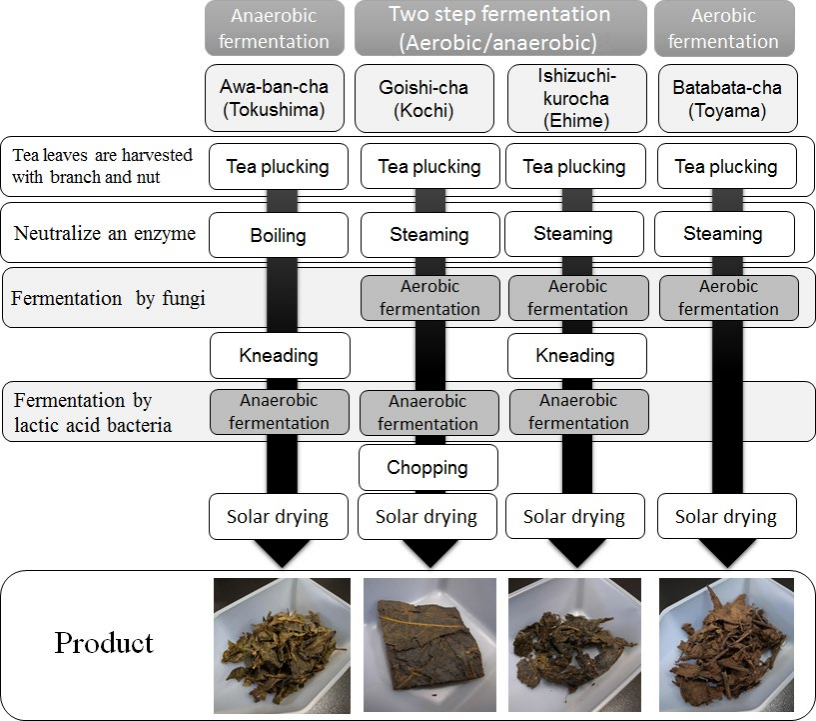
Manufacturing processes of fermented tea. Awa‐bancha is produced by only anaerobic fermentation. Batabata‐cha is produced by only aerobic fermentation. Goishi‐cha and Ishizuchi‐kurocha are produced by two‐step fermentation: aerobic and subsequently anaerobic fermentation. The tea leaves including branch and nut are harvested in early summer. The tea leaves are boiled or steamed for enzyme inactivation and sterilization. In process of Awa‐bancha and Ishizuchi‐kuroha, tea leaves are kneaded for development of anaerobic fermentation. The tea leaves are dried by sunlight after fermentation. We referred several reports for manufacturing process 1–3 and we also heard manufacturing process by producers

There are many kinds of tea in the world. Now, the tea is drunk all over the world. The tea products are classified into three groups; unfermented tea such as green tea, fermented (oxidized) tea such as black tea, and postfermented tea. These teas are made from leaves of Camellia sinensis. In Japan, most common tea is green tea. And black tea and oolong tea are also common tea. According to statistics by Japanese Association of Tea Production, consumed amount of green tea, black tea, and oolong tea in Japan in 2015 was 78846 × 103 kg, 15586 × 103 kg, and 11520 × 103 kg, respectively. Among these three kinds of teas, produce of postfermented tea is most small. The postfermented tea is made by fermentation of tea leaves by microorganisms. Simply stated, the postfermented tea is a pickle of tea leaves. In many cases, the characteristic flavor of fermented tea is produced by fungi and lactic acid bacteria. The most famous postfermented tea is Pu‐erh tea. Traditional fermented tea is made in only two areas in the world. One is the area of Yunnan (China), Myanmar, and northern Thailand. Postfermented teas in this area are Pu‐erh (Yunnan), Lahpetso (Myanmar), and Miang (northern Thailand). The other area is Japan. There are four kinds of traditional postfermented tea in Japan: Ishizuchi‐Kurocha in Ehime, Goishi‐cha in Kochi, Awa‐bancha in Tokushima, and Batabata‐cha in Toyama. Among these postfermented teas, three varieties are made on Shikoku Island. The processes of fermentation and production of these four kinds of postfermented tea are different, respectively (Murakami, 1990; Nakagawa, 1979; Ukeda, 2013) (Figure 1). Ishizuchi‐Kurocha and Goishi‐cha are produced by a two‐stage fermentation process, aerobic fermentation mainly by fungi, and subsequent anaerobic fermentation mostly by lactic acid bacteria. In the process of production of Ishizuchi‐kurocha, tea leaves are kneaded after aerobic fermentation; this is not the case for Goishi‐cha. Awa‐bancha is produced only by anaerobic fermentation by lactic acid bacteria. Batabata‐cha is produced only by aerobic fermentation by fungi. For these reasons, Japanese postfermented teas each have a unique flavor. Recently, postfermented tea attracted attention not only as a favorite beverage but also as a functional beverage (Noguchi, Hamauzu, & Yasui, 2008; Shimamura, Matsuura, Moriyama, Takeda, & Ukeda, 2008; Yoshioka et al., 2013). Two factors can contribute to the functional effect of the postfermented tea. One is the effect mediated by the microorganisms such as lactic acid bacteria. However, association of the fermentation‐related microorganisms to the beneficial effects of the teas remains unclear. The other is the effect of functional compounds present in the tea extract for drinking. Ingredients of postfermented tea are different from those of green tea because of fungal and/or bacterial metabolic activities (Kato et al., 1993, 1995). Therefore, fungi and bacteria involved in the fermentation are important for functional properties of postfermented tea.

In this study, we focused on antioxidant activity of tea. Antioxidant activity of postfermented tea has been studied previously in Goishi‐tea. Goishi‐tea shows superoxide anion scavenging activity of the same magnitude as green tea does (Shimamura et al., 2008). It is also reported that Awa‐bancha has a strong antioxidant activity (Masuda et al., 2013). Nevertheless, there are no studies on the comparison of antioxidant activities among postfermented teas by the same method. Generally, strong antioxidant activity of green tea is derived from catechins, which are some of the polyphenols present in tea leave (Matsuzaki & Hara, 1985). Epicatechin (EC), epigallocatechin (EGC), epicatechin gallate (ECg), and epigallocatechin gallate (EGCg) are known as the catechins of green tea. Among these catechins, EGCg has the strongest antioxidant activity (Nanjo, 1998). Catechins are converted into theaflavin, proanthocyanidin, and theasinensin by polymerization during production of black tea (Nanjo, 2000). Although antioxidant activities have been studied in green tea and black tea varieties, details of antioxidant activity of postfermented tea remain unclear. Chemical changes in catechins in tea leaves during microbial fermentation are poorly understood, and the association of these molecules with the antioxidant activity of postfermented tea remains unknown. Therefore, in this study, we measured concentrations of catechins in postfermented teas and compared the antioxidant activities.

Antioxidant activity of an antioxidant compound depends on the radical‐scavenging ability (Niki & Noguchi, 2000). The simplest method for evaluation of antioxidant activity is DPPH radical‐scavenging activity. Because the DPPH method evaluates the scavenging ability toward artificial radicals per unit of time, the measured antioxidant activity depends on time. This is a demerit of the DPPH method. Although the method of oxygen radical absorbance capacity (ORAC) is a widespread approach to evaluation of antioxidant activity of foods (Cao, Alessio, & Cutler, 1993), this method cannot distinguish whether the cause of the antioxidant activity is the concentration or reactivity of the relevant compounds (Niki, 2010). In this study, the antioxidant activities were measured by two kinds of probes, which have a different rate of reaction with radicals (Takashima et al., 2012). This is an improved method, free of the drawback of the ORAC method. Antioxidant activity in terms of lipid peroxidation was measured by oxygen consumption. Additionally, the metal ion‐reducing ability was evaluated because metal ions generate radicals in the human body. We evaluated all the results on these antioxidative properties and estimated total antioxidant activity of postfermented tea.

## 2 Materials and Methods

### 2.1 Tea materials

All tea leaves were purchased in 2014. Common green tea leaves that were produced in Kagawa, Japan, in 2014 as “Takase‐cha” (Nishimorien, Takamatsu, Japan) were also purchased as a control. The producers of postfermented tea are rare. Ishizuchi‐kurocha is produced only by two companies: Satsuki‐kai and Peace (Saijyo, Ehime, Japan). Ishizuchi‐kurocha was obtained from Satsuki‐kai (referred to as “Ishizuchi‐Kurocha 1” in this study) and Peace (referred to as “Ishizuchi‐Kurocha 2” in this study). Goishi‐cha is produced by some farmers and blended into one product. Goishi‐cha was purchased from Otoyo‐cho Goishi‐cha kyodo‐kumiai (Otoyo, Kochi, Japan). Two kinds of Awa‐bancha were purchased in Kamikatsu, Tokushima, Japan. Batabata‐cha was purchased from Asahi (Asahi, Toyama, Japan).

### 2.2 Analysis of caffeine and polyphenols

Caffeine and polyphenols in tea extracts were determined by high‐performance liquid chromatography (HPLC) based on the method described by Huang et al.(Huang, Inoue, Li, Tanaka, & lshimaru, 2004). One hundred milliliters of boiling water was added to 1.5 g of dry tea leaves with incubation for 5 min. Then, the extract was passed through No. 2 filter paper (Advantec Toyo Kaisha, Ltd., Tokyo, Japan) and a 0.45‐μm membrane filter (Advantec Toyo Kaisha). The filtered extract was applied to a Shimadzu LC‐10A HPLC system (LC‐10AD pump, SPD‐M10AVP PDA detector, and SPD‐10AV UV‐Vis detector; Shimadzu Corporation, Kyoto, Japan). Analysis was performed on a Inertsil ODS‐3 column (4.6 × 150 mm; GL Sciences Inc., Tokyo, Japan). The mobile phase consisted of 0.1% trifluoroacetic acid (TFA) (buffer A) and acetonitrile (buffer B) under the following linear gradient conditions: 10–50% B at 0–30 min, 10% B at 33 min, and 10% B at 40 min. The flow rate was 1.0 ml min^−1^ and column temperature 30°C. Elution was monitored by absorbance at 280 nm. The standard was purchased from Wako Pure Chemical Industries, Ltd. (Osaka, Japan).

### 2.3 Evaluation of copper ion‐reducing activity

It is known that metal ions generate radicals. Thus, the metal ion‐reducing activity of postfermented tea is involved in its antioxidant activity. For evaluation of copper ion‐reducing activity, 100 ml of boiling water was added to 1.5 g of dry tea leaves with incubation for 5 min. The extract and twofold dilution of the extract with distilled water were subjected to the measurements. Reducing activity of the tea extracts toward Cu(II) was measured with the TAC Assay Kit (Metallogenics Co., Ltd., Chiba, Japan) as total antioxidant capacity (TAC) (Campos, Guzmán, López‐Fernández, & Casado, 2009). Cu(II) was reduced to Cu(I) by the antioxidants in the tea extract, and the Cu(I) formed a complex with bathocuproine. The absorbance of the Cu(I)–bathocuproine complex (490 nm) was measured on an Infinite F200 PRO microplate reader (Tecan Group, Ltd., Männedorf, Switzerland).

### 2.4 Evaluation of the radical‐scavenging ability

For evaluation of this ability, 100 ml of boiling water was added to 0.3 g of dry tea leaves with incubation for 5 min. The extract was diluted to an appropriate concentration with distilled water. Details of the method have been described previously (Takashima et al., 2012). The ability of the tea extract to scavenge radicals was assessed on the basis of the effects on the rate of decay of fluorescein (Tokyo Chemical Industry Co., Ltd., Tokyo, Japan) and pyrogallol red (PGR, Aldrich Chemical Company, Inc., Milwaukee, WI, USA) in a reaction with free radicals generated by 2,2′‐azo‐bis(2‐methylpropionamidine) dihydrochloride (AAPH; Wako Pure Chemical Industries). Next, the reaction rates of fluorescein and PGR with free radicals in phosphate‐buffered saline (PBS) were determined by measuring probe decay at 494 and 540 nm by means of a UV‐VIS spectrophotometer (Shinadzu UV1800, Shimadzu Corporation, Kyoto, Japan) equipped with a thermostated cell maintained at 37°C for 2 hr.

The number of radicals (*n*) that were trapped by one molecule of an antioxidant was estimated using the following formula:

(1) $$n=tR_{i}\text{[IH]}$$

where t, [IH], and R_i_ are lag phase time (s), antioxidant concentration (mol\L), and the rate of radical flux from the azo initiator (mol [l·s]^−1^), respectively. In this analysis, R_i_ was estimated by measurement of Trolox (Cayman Chemical, Ann Arbor, MI, USA) because the tea extract contains a mixture of several antioxidants.

### 2.5 Evaluation of antioxidant activity by oxygen consumption

Here, we assessed the antioxidant activity of tea extracts toward oxidation of methyl linolate in micelles of cholic acid (pH 7.4) (Escobar, Salvador, Contreras, & Escamilla, 1990; Umeno et al., 2015). One hundred milliliters of boiling water was added to 0.3 g of dry tea leaves with incubation for 5 min. Next, the extract was passed through a 0.45‐μm membrane filter (Advantec Toyo Kaisha, Ltd.). Micelles were prepared by addition of methyl linolate (0.2 vol%) to 5 ml of 0.1 mol/L cholic acid with vortexing for 2 min. After that, 0.02 ml of the tea extract was added to the micelles with mixing. Then, 5 mmol/L of a radical initiator, AAPH, was added with incubation at 37°C. A decrease in oxygen concentration caused by oxidation of methyl linolate was measured by means of an Oxygen monitor with Clark's oxygen electrode (model 5300, YSI/Nanotech Ltd., Kawasaki, Japan) as a function of time.

### 2.6 Reproducibility

In order to confirm reproducibility, experiments were conducted at least two times.

## 3 Results

### 3.1 Concentrations of caffeine and polyphenols in tea extracts

The extraction conditions recommended by a producer are different for each fermented tea. In this study, however, each postfermented tea was prepared under the same conditions: 100 ml of boiling water was added to 1.5 g of tea leaves with incubation for 5 min. Concentrations of caffeine and catechins per 100 ml of each tea extract are shown in Table 1. Compared with green tea as a control unfermented tea, caffeine content of postfermented tea was low. Catechin content of postfermented tea was also lower than that of green tea. Among the postfermented teas, the concentration of all catechins was the highest in Ishizuchi‐kurocha. In particular, the concentration of EGC in Ishizuchi‐kurocha was high. On the other hand, Batabata‐cha hardly contained catechins. The concentration of catechins was the lowest in Awa‐bancha. As for Ishizuchi‐kurocha and Awa‐bancha, two kinds of tea leaves that were produced by different companies were analyzed. Differences in caffeine and catechins content between the leaves from the two producers were small. Concentrations of caffeine and catechins depend on the fermentation process. As for Goishi‐cha and Batabata‐cha, tea leaves produced by some producers were blended into one product.

**Table 1:** Concentration of caffeine and catechin in fermented tea

	Fermentation method	Caffeine	EGC	EGCg	EC	GCg	Ecg	Total catechins
Green tea	Nonfermentation	43.7^a^	24.5^a^	45.9^a^	9.5^a^	0.9^a^	6.9^a^	87.8^a^
Goishicha	Two‐step (aerobic/anaerobic)	11.6^b^	6.5^c^	0.4^b^	0.2^b^	Trace	Trace	7.1^c^
Ishizuchi‐Kurocha 1		13.9^b^	16.0^b^	2.0^b^	1.9^b^	0.1^a^	0.1^b^	20.1^b^
Ishizuchi‐Kurocha 2		15.7^b^	19.5^b^	1.0^b^	4.8^ab^	0.1^a^	Trace	25.3^b^
Awa‐Bancha 1	Anaerobic	6.1^bc^	7.0^c^	Trace	0.6^b^	0.2^a^	0.1^b^	7.9^c^
Awa‐Bancha 2		3.8^c^	7.8^c^	0.2^b^	1.1^b^	0.1^a^	0.5^b^	9.7^c^
Batabata‐cha	Aerobic	7.8^bc^	0.4^d^	Trace	0.1^b^	Trace	Trace	0.4^d^

EC, Epicatechin; EGC, epigallocatechin; EGCg, epigallocatechin gallate

Tea extracts were eluted by 100 ml of boiling water from 1.5 g of tea leaves for 5 min.

Data shown represent mean of five experiments (*n* = 5).

Means in the same column with different superscript letters are significant at *p* < .05.

### 3.2 Evaluation of antioxidant activity of postfermented tea

Antioxidant activities of postfermented teas were measured by three methods. The reducing efficiency of tea extracts against Cu(II) was measured first (Table 2). The reducing efficiency was the strongest in green tea. In ascorbate equivalents, the antioxidant activity of green tea was 2.6 mmol/L. The antioxidant activity of Ishizuchi‐kurocha 1 was 2.1 mmol/L. The antioxidant activities of Goishi‐cha, Awa‐bancha, and Ishizuchi‐kurocha 2 were approximately 1.2–1.9 mmol/L. In contrast, the antioxidant activity of Batabata‐cha was 0.1 mmol/L. Overall, the antioxidant activity as copper ion‐reducing efficiency of the postfermented teas was ranked as follows: green tea > Ishizuchi‐kurocha = Goishi‐cha > Awa‐bancha > Batabata‐cha.

**Table 2:** Copper‐reactive capacity of fermented tea

		Antioxidant activity^a^		Copper reductive capacity (mmol/L)	
	Fermentation method	Undiluted solution	Doubling dilution	Undiluted solution	Doubling dilution
Green tea	Nonfermentation	ND	2.61^a^	ND	5.22
Goishicha	Two‐step (aerobic/anaerobic)	ND	1.92^b^	ND	3.84
Ishizuchi‐ Kurocha 1		ND	2.14^c^	ND	4.28
Ishizuchi‐ Kurocha 2		2.63	1.43^d^	5.26	2.86
Awa‐Bancha	Anaerobic	1.53	0.81^e^	3.06	1.62
Batabata‐cha	Aerobic	0.17	0.10^f^	0.34	0.2

a Estimated as equivalent to ascorbic acid (mM).

ND means that the data could not be determined because the absorbances were off‐scale high.

Means in the same column with different superscript letters are significant at *p* < .01. (ANOVA, Tukey).

**Figure 2:**
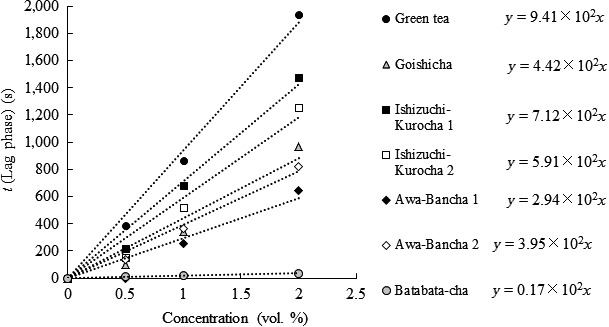
Radical trapping ability of fermented tea. The ability of the diluted tea extract to scavenge radicals was assessed by means of the effects on the rates of decay of fluorescein in a reaction with free radicals generated by AAPH. The reaction rates of fluorescein with free radicals in phosphate‐buffered saline (PBS) were determined by measuring probe decay at 494 nm on a UVVIS spectrophotometer equipped with a thermostated cell maintained at 37°C for 2 hr. The calculation method is described in Materials and Methods

Next, antioxidant activity was evaluated as radical‐scavenging capacity. A fluorescent probe and a radical initiator were mixed with each tea extract and temporal decay of fluorescence as a result of oxidation of the probe was measured. Because reactivity of fluorescein with radicals is low, if antioxidants exist in the solution, oxidation of fluorescein by radicals is inhibited and fluorescence is retained. Therefore, antioxidant activity of the tea extracts was evaluated by means of time before decay of fluorescence (lag phase). The lag phase determined for each tea extract is shown in Figure 2. The slope of linear approximation is proportional to radical‐scavenging capacity. Green tea showed the highest radical‐scavenging capacity. Ishizuchi‐kurocha, Goishi‐cha, and Awa‐bancha also showed radical‐scavenging capacity. On the other hand, Batabata‐cha hardly showed any radical‐scavenging capacity in this assay. Overall, radical‐scavenging capacity of the postfermented teas was ranked as follows: green tea > Ishizuchi‐kurocha > Goishi‐cha > Awa‐bancha > Batabata‐cha. This order is similar to the ranking of copper ion‐reducing efficiency.

**Figure 3:**
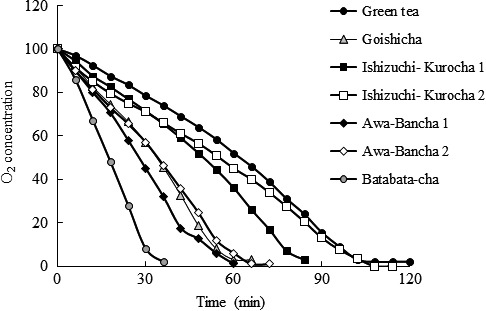
Evaluation of inhibition of oxygen consumption by fermented tea. Antioxidant activity of the tea extracts was assessed towards oxidation of methyl linolate in micelles of cholic acid (pH 7.4). The tea extract and 5 mM AAPH were added to the micelles with incubation at 37°C. A decrease in oxygen concentration caused by oxidation of methyl linolate was measured by means of an Oxygen monitor with Clark's oxygen electrode as a function of time

Additionally, radical‐scavenging ability was measured using PGR, which is a high‐reactivity probe for radicals. All postfermented tea extracts showed a certain radical‐scavenging rate, but there were no significant differences. The effect was not concentration dependent. Moreover, we examined antioxidant activity of the postfermented tea extracts by means of oxygen consumption. The radical initiator, AAPH, was added to a mixture of methyl linolate, cholic acid, and a tea extract, and then the oxygen consumption was measured. Although oxygen in these mixtures is consumed by AAPH, if an antioxidant compound is present, the consumption of oxygen is reduced. Results are shown in Figure 3. Green tea inhibited oxygen consumption as efficiently as it reduced copper ions and scavenged radicals. Overall, the ranking of inhibitory effects on oxygen consumption among the tea extracts was as follows: green tea > Ishizuchi‐kurocha > Goishi‐cha = Awa‐bancha > Batabata‐cha.

## 4 Discussion

In this study, we measured catechin content of Japanese traditional postfermented teas and evaluated their antioxidant activities. It is reported that catechins are involved in the antioxidant activity of green tea (Matsuzaki & Hara, 1985). The concentration of catechins in the postfermented teas was 29% of the concentration in green tea at a maximum (Ishizuchi‐kurocha), and 0.5% at a minimum (Batabata‐cha). In Ishizuchi‐kurocha, EGC content was high. Although catechin content of the postfermented teas was only ~30% relative to green tea at a maximum, most of our postfermented teas showed sufficient antioxidant activity. Green tea had strongest antioxidant activity as measured by three methods for quantification of antioxidant properties. Antioxidant properties of Ishizuchi‐kurocha were comparable to those of green tea. On the other hand, Batabata‐cha hardly showed any antioxidant properties. Strength of antioxidative activities of each postfermented tea was ranked approximately as follows: green tea > Ishizuchi‐kurocha > Goishi‐cha > Awa‐bancha > Batabata‐cha, regardless of the evaluation method. The tea extracts showed not only radical‐scavenging activity but also metal ion‐reducing activity and inhibition of lipid peroxidation. A metal ion causes formation of radicals in vivo, and lipids are most susceptible to oxidative stress in the human body (Nordmann, Ribière, & Rouach, 1987; Valko, Morris, & Cronin, 2005). Therefore, postfermented tea may exert a wide range of antioxidative actions in vivo. Because living organisms are chemically inhomogeneous and complicated, multifaceted evaluation is important for assessment of in vivo antioxidant activity of postfermented tea. As shown in Figure 1, Ishizuchi‐kurocha and Goishi‐cha are made by two‐step fermentation, aerobic fermentation by fungi and subsequent anaerobic fermentation by lactic acid bacteria. On the other hand, Awa‐bancha is made only by anaerobic fermentation by lactic acid bacteria. Batabata‐cha is made only by aerobic fermentation by fungi. It is possible that these fermentation procedures affect the antioxidant activity of postfermented tea. Among the four kinds of postfermented tea, antioxidant activity of Batabata‐cha, which is made only by aerobic fermentation, was very low. In contrast, antioxidant activity was strong in Awa‐bancha, which is made only by anaerobic fermentation. Furthermore, antioxidant activities of Ishizuchi‐kurocha and Goishi‐cha were stronger than those of the other two kinds of postfermented tea. These observations suggest that anaerobic fermentation by lactic acid bacteria is important for antioxidant activity of postfermented tea. In this study, although we did not identify the antioxidant compounds contributing to the antioxidant activity of each postfermented tea, concentrations of catechins are low in all kinds of postfermented teas. Tea extracts of postfermented tea show a bistered or brown color. Therefore, the antioxidant compounds of postfermented tea may be polymers of catechins formed during anaerobic fermentation. On the other hand, there are many differences in production process other than fermentation between green tea and postfermented tea. First, young leaves for new green tea are harvested from late spring to early summer in Japan, from April to June, like in Japanese children's song “*Chatsumi*” (tea picking). Among new green tea leaves, only young leaves are harvested. In contrast, for postfermented tea, tea leaves are harvested from late June to early August. For postfermented tea, grown leaves including branches and nuts are harvested. Therefore, there is a possibility that the concentration of caffeine and catechins during picking of tea leaves is already different between green tea and postfermented tea before the fermentation process. On the other hand, it is reported that contents of catechins were reduced in Awa‐bancha and Ishizuchi‐kurocha during fermentation (Kato et al., 1993, 1995). In this study, we compared concentrations of caffeine and catechins among tea extracts as a “final product.” Changes in caffeine and catechins content during a fermentation process were not analyzed. This is a task for a future study. Of course, postfermented tea is a favorite beverage, and its unique taste and flavor are important. Nevertheless, sometimes consumers select foods and drinks based on physiological effects such as antioxidant properties. Antioxidant activity is one of the beneficial properties, which contributes to a favorable brand image of foods. Strong antioxidant activity and low caffeine content are hailed as good properties of postfermented teas. Although Batabata‐cha hardly showed any antioxidant activity, it is not inferior to other postfermented teas. From another perspective, caffeine content is lower than in Ishizuchi‐kurocha and Goichi‐cha. This is another good property for a favorable brand image. Thus, postfermented teas have a potential for favorable physiological effects. The results suggest that anaerobic fermentation process is beneficial at least for antioxidative activity.

## Conflict of Interest

None declared.
